# Membrane frizzled-related protein gene–related ophthalmological syndrome: 30-month follow-up of a sporadic case and review of genotype-phenotype correlation in the literature

**Published:** 2012-10-26

**Authors:** Alberto Neri, Rosachiara Leaci, Juan C. Zenteno, Cristina Casubolo, Elisabetta Delfini, Claudio Macaluso

**Affiliations:** 1 Ophthalmology, University of Parma, Parma, Italy; 2 Department of Biochemistry, Faculty of Medicine, UNAM, Mexico City, Mexico; 3 Department of Genetics-Research Unit, Institute of Ophthalmology “Conde de Valenciana,” Mexico City, Mexico

## Abstract

**Purpose:**

To report a new sporadic case of membrane frizzled-related protein gene (*MFRP*)-related syndrome with a 30-month follow-up, and to review the literature for genotype-phenotype correlation in MFRP mutations.

**Methods:**

A complete ophthalmological evaluation was performed at presentation and 30 months later, including best-corrected visual acuity test, slit lamp examination, fundoscopy, kinetic perimetry, electroretinography, fundus imaging (color, red-free, and autofluorescence), and morphologic-biometric analysis of the eye structures with an optical biometer, anterior-segment optical coherence tomography, retinal optical coherence tomography, and a confocal scanning laser for optic nerve head study. Polymerase chain reaction amplification of DNA obtained from peripheral blood lymphocytes and nucleotide sequencing of the complete *MFRP* gene were performed. The literature on cases of posterior microphthalmos and retinitis pigmentosa associated with *MFRP* mutations was reviewed.

**Results:**

A 33-year-old female patient presented with posterior microphthalmos, retinitis pigmentosa with patches of retinal pigmented epithelium atrophy and scarce pigment mobilization, foveoschisis, and optic nerve drusen. After 30 months, progression of rod-cone retinal degeneration was detected. One obligate carrier showed a normal eye phenotype. A homozygote mutation in the *MFRP* gene (c.492delC), predicting a truncated protein (P166*fs*X190), was identified with genetic analysis. To our knowledge, 17 cases of *MFRP*-related syndrome have been reported in the literature, including the patient described herein. The phenotype of the syndrome, expressivity, and age of onset varied among and within the affected families. However, all patients sharing homozygous mutation c.492delC (alternatively named c.498delC) showed a complete phenotype (including foveoschisis and optic nerve head drusen), and similar fundus characteristics.

**Conclusions:**

A new sporadic case of *MFRP*-related syndrome is reported. Review of the literature showed variability in the phenotype, but initial elements of genotype-phenotype correlation have been identified in patients sharing the mutation of the present case.

## Introduction

Several reports correlate mutations of membrane frizzled-related protein gene (*MFRP*, OMIM 606227) with a recessively inherited ocular syndrome characterized by the association of posterior microphthalmos (PM), retinitis pigmentosa (RP), foveoschisis, and optic nerve head (ONH) drusen [1–4]. *MFRP* mutations had been previously related to a different ocular phenotype, characterized by autosomal-recessive nanophthalmos without fundoscopic or electrophysiologic signs of retinal dystrophy [5,6]. The literature showed high variability of the clinical phenotype of *MFRP* mutations among and within affected families, and attempts to correlate specific gene mutations with different clinical phenotypes have failed [2].

We review the existing literature regarding *MFRP*-related syndrome, and describe a new sporadic case with a 30-month follow-up caused by a homozygote 1-bp deletion in exon 5 of the *MFRP* gene, shortening a set of seven consecutive cytosines running from position 492 to 498. This mutation, predicting a truncated protein (P166*fs*X190), has been already described in homozygosis [2,3] and in compound heterozygosis [2,4,6] in previous publications, where the mutation was named alternatively c.492delC or c.498delC. The present study supplies further elements to the characterization of this rare and heterogenic syndrome.

## Methods

A 33-year-old female patient and a healthy carrier (male, eight-year-old son of the patient) were recruited. The proband was referred to our department by a general ophthalmologist for diagnostic work-up due to the findings of extreme hyperopia and reduced visual acuity. Other relatives were not available for examination. The patient expressed informed consent for the study, and the participants were treated in accordance with the principles of the Declaration of Helsinki. A complete ophthalmological evaluation was performed at presentation and 30 months later, including best-corrected visual acuity (BCVA) testing with early treatment diabetic retinopathy study (ETDRS) charts, pupil examination, applanation tonometry (Goldmann tonometer), kinetic perimetry (Goldmann perimeter), funduscopy, and ocular ultrasonography. Corneal curvature (keratometry), eye axial length, and corneal diameter (white-to-white distance) were measured with an IOL-Master (Carl Zeiss Inc., Jena, Germany). A high-resolution swept-source anterior segment optical coherence tomography device (CASIA OCT; Tomey Co., Nagoya, Japan) was used to obtain three-dimensional visualization and measurements of the eye anterior segment. Color pictures of the retina were acquired using a mydriatic fundus camera (Topcon 50X, Tokyo, Japan), while fundus infrared (IR), red-free (RF), and autofluorescence (AF) images were obtained with a scanning laser ophthalmoscope (SLO; HRA2; Heidelberg Engineering, Heidelberg, Germany). AF images are particularly useful in the present case as the most frequent sources of autofluorescence in the fundus are the lipofuscin accumulated within the retinal pigmented epithelium (RPE), hence facilitating the visualization of RPE atrophic areas (appearing as hypo-autofluorescent areas), and the calcific hyaline bodies forming ONH drusen (appearing as hyper-autofluorescent areas). Optic nerve head analysis was performed with a confocal SLO (HRT, Heidelberg Engineering). Optical coherence tomography of the retina was performed using a spectral domain OCT (Cirrus HD-OCT, Carl Zeiss Inc.). Full field electroretinograms (ERGs) were recorded with conjunctival electrodes (H-K loops) with a Ganzfeld stimulator (Espion, Diagnosys, Lowell, MA), according to International Society of Clinical Electrophysiology of Vision (ISCEV) standards [7]. Blood was obtained via a peripheral vein puncture, and preserved at −70 °C before use. Genomic DNA was obtained from peripheral blood lymphocytes according to standard methods and genetic analysis of *MFRP* was performed as described by Crespí et al. 2008 [3]. Sequence changes were verified by sequencing both DNA strands.

Review of the literature about *MFRP*-related syndrome was performed on PubMed (NLM) including the described cases of posterior microphthalmos and retinitis pigmentosa associated with *MFRP* mutations. Cases with *MFRP* mutations without evidence of retinal degeneration were not included.

## Results

Appendix 1 shows the main features of 17 cases of *MFRP*-related PM and RP, including the one described herein and those we found in the literature [1–4]; in two additional cases with PM and RP, genetic analysis failed to identify *MFRP* mutations [8].

The clinical features of our patient are reported in Table 1 and Table 2. The patient was born in France to non-consanguineous parents (French mother, Tunisian father). She has been complaining of visual acuity problems since early childhood, due to high hyperopia. She denied the existence of any similar case of eye disease among her relatives, as well as of any consanguineous marriage in the family. At the time of recruitment (2009), the patient was 33 years old; she had noticed a slowly progressive decrease in visual acuity in the last three or four years. She did not notice night blindness or photophobia. BCVA was 20/100 with +17D spectacle correction in both eyes. Intraocular pressure was 15 mmHg in both eyes, and pupil reflexes were normal. Slit lamp examination did not reveal anomalies of the anterior segment of the eye. Kinetic perimetry revealed mild modifications, with moderate isopter constriction.

**Table 1:** Biometric measurements of the patient eyes.

**Measurement**	**Method**	**Right eye**	**Left eye**
Axial length	IOL-Master	15.85 mm	15.91 mm
Horizontal corneal diameter (White-to-white distance)	IOL-Master	11.9 mm	11.9 mm
Anterior corneal curvature (Keratometry) r-min (K-steep)	AS-OCT	6.88 mm (49.0D)	6.94 mm (48.6D)
Anterior corneal curvature (Keratometry) r-max (K-flat)	AS-OCT	7.16 mm (47.1D)	7.19 mm (46.9D)
Anterior corneal curvature (Keratometry) avg-r (avg-K)	AS-OCT	7.02 mm (48.1D)	7.07 mm (47.8D)
ACD	AS-OCT	3.41 mm	3.41 mm
CCT	AS-OCT	514 µm	506 µm
AR-AR	AS-OCT	11.5 mm	11.5 mm
Lens thickness	AS-OCT	5.70 mm	5.65 mm
Superior irido-corneal angle	AS-OCT	36.4°	21.0°
Nasal irido-corneal angle	AS-OCT	29.2°	20.5°
Inferior irido-corneal angle	AS-OCT	34.7°	24.5°
Temporal irido-corneal angle	AS-OCT	30.5°	27.4°
Optic disc area	HRT	1.511 mm^2^	1.582 mm^2^
Cup/disk area ratio	HRT	0.004	0.214

Anterior corneal curvature abbreviations: r-min, r-max and r-avg are the minimum, maximum and average radius of curvature of the anterior corneal surface, respectively; the corresponding keratometric values, expressed in diopters (D), are indicated in parenthesis. ACD: anterior chamber depth (distance from the corneal epithelium to the lens anterior surface). CCT: central corneal thickness. AR-AR: distance between temporal and nasal angular recesses measured in horizontal AS-OCT scans of the anterior segment of the eye. Lens thickness is measured along the fixation axis in vertical sections of the lens obtained with AS-OCT. Irido-corneal angles were measured with a dedicated function of the AS-OCT on horizontal and vertical OCT B-scans of the anterior segment of the eye.

**Table 2:** Evolution of main clinical parameters over a thirty-month follow-up.

**Time**	**Visual acuity ETDRS a.c.**	**Isopter horizontal diameter** **Kinetic perimetry**		**Foveal thickness SD-OCT**
		IV-4	I-4	
Presentation	20/100 RE	135° RE	67° RE	575 µm RE
	20/100 LE	130° LE	60° LE	545 µm LE
30 month	20/125 RE	120° RE	65° RE	591 µm RE
	20/125 LE	110° LE	57° LE	518 µm LE

ETDRS a.c.: Early Treatment Diabetic Retinopathy Study acuity charts. SD-OCT: spectral domain OCT.

Posterior microphthalmos with axial length of 15.85 mm and 15.91 mm in right and left eye, respectively, was the cause of the high hypermetropia, while the anterior segment appeared normally developed. Biometric measurements are reported in Table 1.

**Figure 1:**
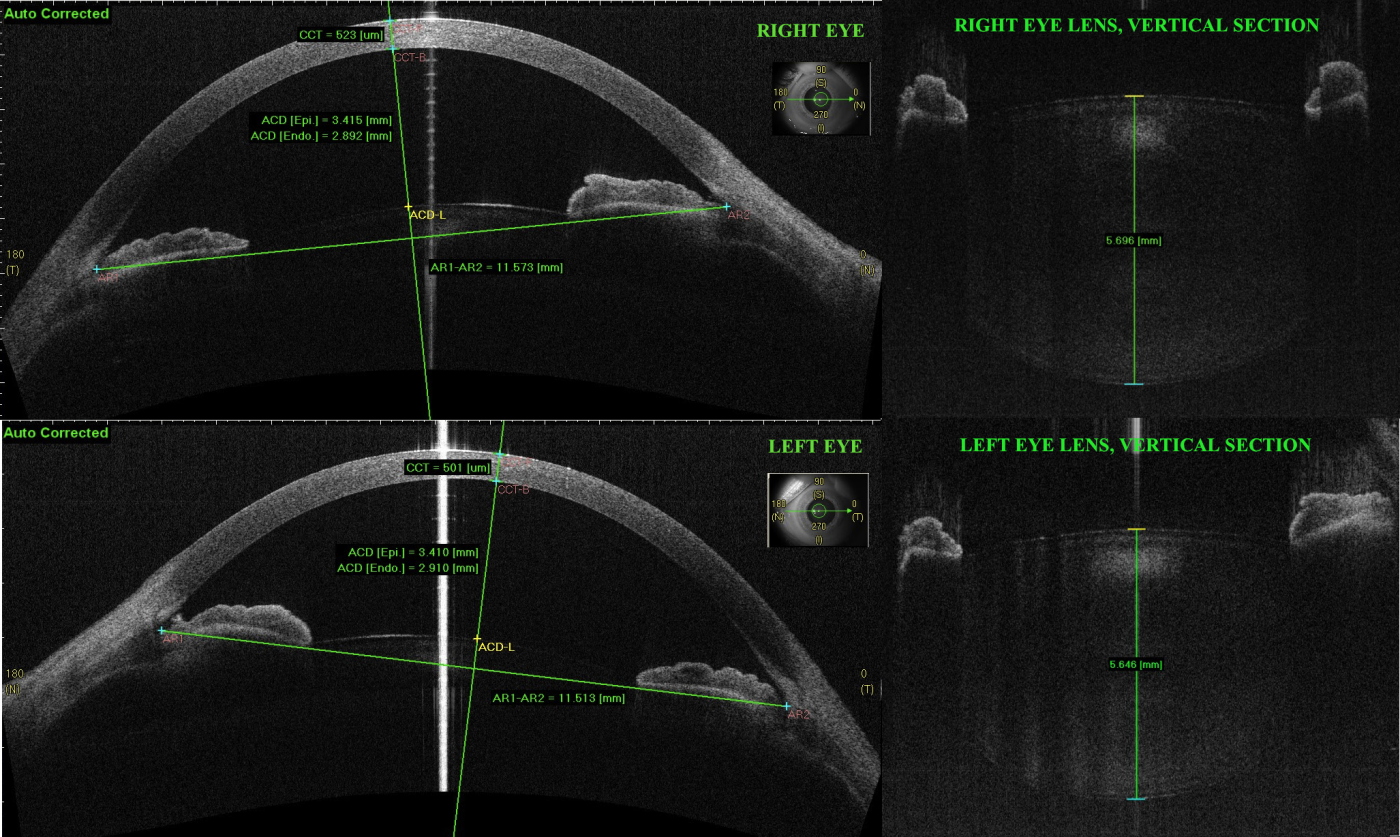
Analysis of the anterior segment of the patient's eyes at presentation with spectral-domain anterior segment optical coherence tomography. The images are automatically corrected with the anterior segment optical coherence tomography (AS-OCT) software accounting for the optical effect of the anterior and posterior surfaces of the cornea. On the left: horizontal B-scans of the anterior chamber. CCT: central corneal thickness; ACD: anterior chamber depth, ACD [epi]: distance from the corneal epithelium to the lens anterior surface; ACD [endo]: distance from the corneal endothelium to the lens anterior surface; AR1-AR2: anterior chamber width, from temporal to nasal angular recess. On the right: vertical B-scans centered on the lens. Lens thickness is measured along the fixation axis.

**Figure 2:**
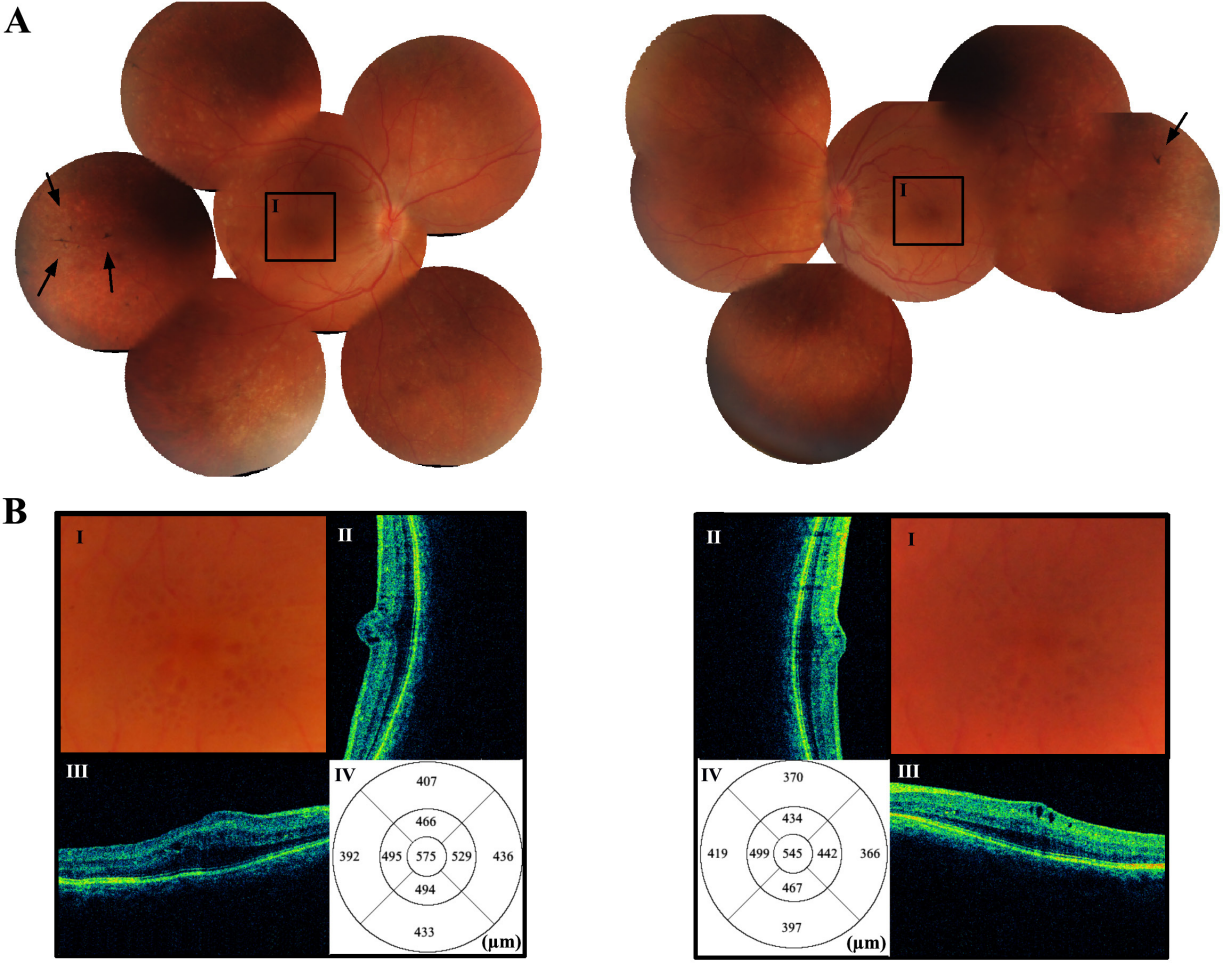
Retinography and optical coherence tomography imaging taken at presentation (right eye on the left panel). No significant morphological modifications were detected at the 30-month follow-up examination. **A**: Color pictures of the patient's fundus show small optic discs and retinal pigmented epithelium mottling and atrophy, appearing as pale yellowish-white retinal spots similar to flecks. There are a few bone spicule-like deposits at the temporal periphery (arrows). Square insets highlight the macula, which is shown enlarged in the lower panel **I**: **B:** Color pictures of the patient's macula **I**: vertical and horizontal optical coherence tomography (OCT) scans of the retina passing at the fovea (II, III), and Early Treatment Diabetic Retinopathy Study (ETDRS) maps of macular thickness measured with spectral domain OCT (IV). Multiple cystic spaces in the retinal inner layers are present at the fovea, with increased foveal thickness in both eyes.

**Figure 3:**
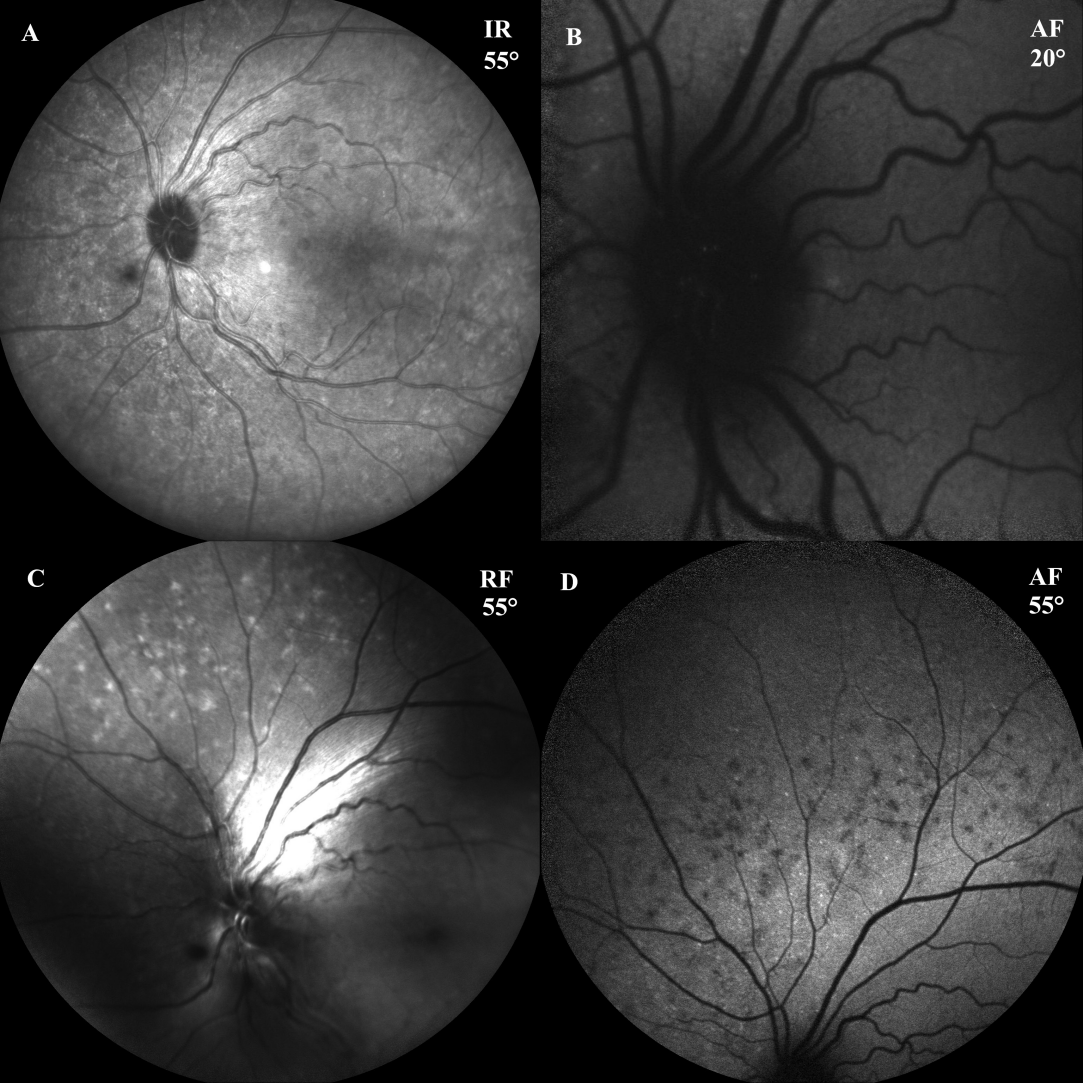
Fundus pictures obtained at presentation with a scanning laser ophthalmoscope. **A**: 55° infrared (IR) image centered at the posterior pole. **B**: 20° retinal auto-fluorescence (AF) imaging of the optic nerve head (OHN), showing some small hyper-auto-fluorescent spots corresponding to ONH drusen. **C**: 55° red-free (RF) image: the areas of retinal pigmented epithelium (RPE) mottled atrophy appear as brighter spots. **D**: 55° AF image: the same RPE atrophic spots shown in **C**: appear darker in AF imaging.

Figure 1, Figure 2, and Figure 3 show the anterior segment and retinal imaging data obtained at presentation, while no significant morphological modifications were detected at the 30-month follow-up examination. Fundoscopy revealed small optic nerve heads (Table 1, Figure 2, and Figure 3), macular alterations consisting of multiple cystic lesions at the fovea (Figure 2B), and RPE mottling and atrophy, appearing as pale yellowish-white retinal spots similar to flecks, diffuse mainly at the posterior pole and at the equator (Figure 2A). The RPE atrophic spots were easily detectable with red-free scanning laser imaging as brighter spots (Figure 3C), while they appeared dark with fundus autofluorescence imaging (Figure 3D), in keeping with their RPE atrophic origin. Pigment mobilization was scarce, with a few bone spicule-like pigmentary deposits at the temporal periphery (Figure 2A, arrows). No sign of annular fundus autofluorescence was detected. Optic nerve head drusen was identified in both eyes with fundus autofluorescence as hyper-autofluorescent spots inside the optic disc area (Figure 3B). Macular OCT (Figure 2B) showed diffuse macular thickening and outer layer cystic foveal spaces, with abolished foveal slope and augmented retinal foveal thickness (575 µm and 545 µm in the right and left eyes, respectively).

**Figure 4:**
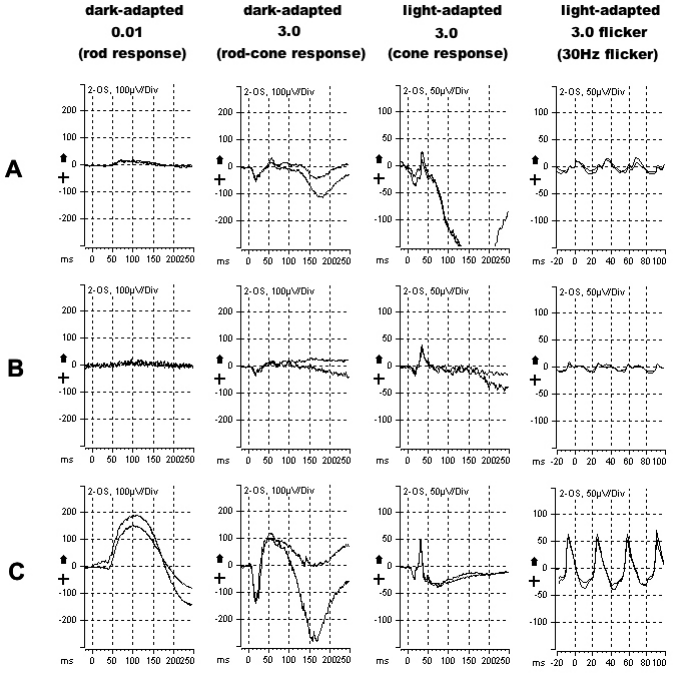
Electroretinograms of the patient at baseline **A**: and after 30 months **B**: compared to a normal control **C**: showing severely reduced scotopic and photopic responses, with evidence of progression over the follow-up period, in keeping with the diffuse rod-cone photoreceptor degeneration. The four columns represent different categories of electroretinogram (ERG) response, labeled according to the names suggested by the ISCEV standard [7]. Each graph shows two repetitions of the same response, all recorded from the left eye.

The ERG at presentation (Figure 4A) showed severely reduced but recordable scotopic and attenuated photopic responses, in keeping with the diffuse rod-cone photoreceptor degeneration. The son of the patient, an obligate carrier of the mutated gene, showed normal eye morphometry, and was emmetropic. His uncorrected visual acuity was 20/16.

Thirty months later (Table 2), the patient reported a reduction in visual acuity and progressively worsening night-blindness. BCVA had decreased to 20/125 in both eyes. Optical coherence tomography of the macula showed substantially unchanged anatomic alterations and stable retinal foveal thickness in both eyes. Kinetic perimetry showed minimal progression in visual field constriction. Repetition of ERG testing showed further reduction of all ERG components, with the rod response becoming barely detectable, confirming the progression of the rod-cone retinal degeneration (Figure 4B). The ophthalmologic examination of the carrier was unmodified.

**Figure 5:**
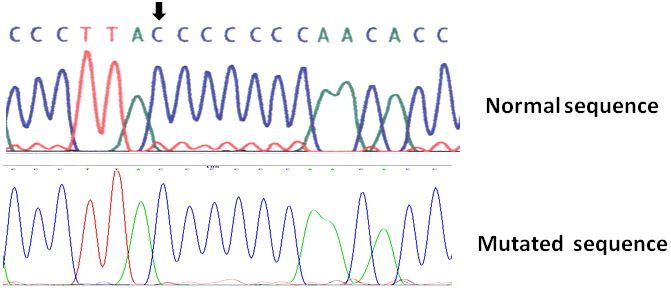
Partial sequence analysis of membrane frizzled-related protein gene (*MFRP*) gene exon 5 in the MFRP-related ophthalmological syndrome. Normal sequence from the control DNA is shown in the top panel. A homozygous **C**: deletion at nucleotide position 492, named c.492delC (codon 164) in DNA from the affected individual is shown in the bottom sequence. The deleted nucleotide is highlighted with an arrow in the normal DNA. This mutation predicts a truncated MFRP protein (P166fsX190).

Genetic sequencing of the *MFRP* gene identified a homozygote 1-bp deletion (c.492delC) in exon 5 (Figure 5). This mutation originates a shift of the open reading frame from residue proline 166 and predicts a premature truncation of the protein, 25 codons downstream (P166fsX190). No additional deleterious mutations were observed in any of the remaining *MFRP* exons in the patient's DNA.

## Discussion

Nanophthalmos or simple microphthalmos is a congenital defect characterized by an abnormally small ocular globe (i.e., with axial length at least two standard deviations below age-similar controls) without associated anatomic defects [9]. The short axial length causes high hyperopia, and the shallow anterior chamber is associated with an elevated risk of angle-closure glaucoma. Spontaneous or post-surgical uveal effusion syndrome is a described complication of nanophthalmos [10,11].

PM is an uncommon variety of nanophthalmos in which an abnormal, underdeveloped posterior segment of the eye coexists with a normally developed anterior segment [12,13]. PM is prevalently sporadic, but familial cases with autosomal-recessive inheritance pattern have been reported [12]. Associated posterior segment anomalies, including elevated papillomacular retinal fold, chorioretinal folds, pigmentary retinopathy, uveal effusion syndrome, and macular hole have been reported [14,15]. The disproportion between the short axial length and the normal curvature of the cornea and lens optical surfaces results in extremely high hyperopia, while the association with glaucoma is not typical, since the anterior chamber structures are normal.

Different authors in recent years described a recessively inherited ocular syndrome characterized by the association of PM and RP, in some instances associated with foveoschisis and ONH drusen [1–4,8]. This rare syndrome was consistently correlated with homozygote mutations in *MFRP*, OMIM 606227 [1–4]. However, other genes could be responsible for a similar phenotype, as a recent work describing two Indian siblings affected by PM and RP failed to identify mutations in the *MFRP* gene [8]. MFRP gene mutations had been previously found also in association with another ocular phenotype, consisting of a form of autosomal-recessive nanophthalmos with shallow anterior chamber and high corneal curvature, but without clear-cut evidence of retinitis pigmentosa [5,6].

Further advance in the knowledge of MFRP molecular structure and function came from the study of an animal model of *MFRP* mutations, the retinal degeneration 6 mouse (rd6 mouse). rd6 mice present progressive retinal degeneration with characteristics resembling human flecked dystrophies (Stargardt disease, fundus flavimaculatus, retinitis punctata albescens), in the absence of nanophthalmos or pathological hyperopia [16–18]. As already pointed out [6], the lack of the latter anomalies in the mouse model might be accounted for by the profound developmental differences between the mouse and the primate eye.

The *MFRP* gene encodes for a membrane protein mainly expressed in the RPE cells, where it is localized at the cells apical membrane [19,20]. The C-terminal domain of MFRP was correlated with the frizzled (Fz) family of proteins, which are involved in cellular determination as receptors for wingless (Wnt) proteins. The Wnt signal pathway, in turn, had been previously related to retinal dystrophies [21]. In human and mouse, *MFRP* is expressed as a dicistronic transcript together with another gene, *C1QTNF5*/*CTRP5* (complement-1q tumor necrosis factor-related protein 5), which is colocalized with MFRP in RPE and ciliary bodies and whose mutation was shown to cause late-onset retinal degeneration [17,18,20,22,23]. However, in the rd6 mouse null mutation of *Mfrp* did not show any influence on the expression and cellular localization of C1qtnf5 [18]. A recent human study revealed the presence of a functional promoter for the *C1QTNF5*/*CTRP5* gene located 5′ of its start site [24]. Further study on the regulation of *C1QTNF5*/*CTRP5* gene transcription will determine whether *CTRP5* and *MFRP* are functionally dicistronic, and whether *CTRP5* plays a role in determining the ocular phenotype of *MFRP*-related syndrome.

To our knowledge, 17 cases of *MFRP*-related syndrome have been reported in the literature, including the patient described herein (Appendix 1). The phenotype of the syndrome varies among and within the affected families, and expressivity and age of onset varied among patients (Appendix 1).

Most cases (11/17) with the “complete” phenotype, including PM, RP, ONH drusen, and foveoschisis, may be distinguished from cases with “incomplete” phenotypes (6/17), in which both foveoschisis and ONH drusen (four cases) or only ONH drusen (two cases) was absent. Moreover, some cases showed advanced RP characterized by conspicuous pigment mobilization, bone spicule-like retinal pigmentation, and extensive concentric atrophy, while other cases were characterized by scarce pigment mobilization and clumping, no or few bone spicule-like deposits, and less severe RPE atrophy. Although angle closure glaucoma is not a typical feature of *MFRP*-related syndrome, development of glaucoma during adolescence was reported in two cases [3]. Nonetheless, anterior chamber depth does not appear as a consistent feature in patients with MFRP-related syndrome [1–4].

In our patient, the homozygote 1-bp deletion c.492delC was identified. The same mutation had been previously identified in patients with *MFRP*-related syndrome in homozygosis [2,3] and in compound heterozygosis with other nonsense [2,4] or missense mutations [6]. All cases carrying the c.492delC mutation either in homozygosis [2,3] or in compound heterozygosis with other nonsense mutations [2,4] shared the complete phenotype, including PM, RP, foveoschisis, and ONH drusen, although this last feature was absent in one case [2]. An additional family with the c.492delC mutation in compound heterozygosis with missense mutation I182T showed a milder phenotype, with patches of hypopigmentation in the peripheral retina and initial functional defects in keeping with the presence of initial photoreceptor degeneration [6].

Fundoscopy in our case was similar to that described by Zenteno et al. [4] in two Mexican siblings, affected by the same c.492delC *MFRP* mutation in compound heterozygosis with another nonsense mutation: there were multiple irregular retinal yellowish-white spots of retinal hypopigmentation, mainly diffuse at the posterior pole, foveoschisis, and optic nerve drusen. In our case there was minimal intraretinal pigmentation, with a few Bone spicule-like deposits at the periphery, which were not present in the cases described by Zenteno et al. [4]. The patches of retinal depigmentation are also similar to those described by Sundin et al. [5,6] in patients showing borderline evidence of retinal degeneration. Moreover, in the present case progression of the rod-cone retinal degeneration associated with the disease was demonstrated by the worsening of the ERG and visual field tests repeated after 30 months, as well as by the onset of new symptoms (night blindness).

Carriers of *MFRP* mutations have been examined in different pedigrees, reporting in all cases normal eye phenotype and no tendency to hyperopia [1–4]. We also examined one obligate carrier, finding normal eye phenotype and emmetropic refraction. A previous study excluded a role for *MFRP* in determining physiologic hyperopia [25].

*MFRP* mutations could cause a spectrum of retinal alterations, including patients with aggressive retinal degeneration starting in infancy as well as patients with no symptoms in elderly age. Although in general *MFRP*-related syndrome showed significant variability even within affected families, the present case and the others carrying the mutation c.492delC, either in homozygosis or compound heterozygosis with other nonsense mutations appeared to share a more consistent phenotype. In fact, all these patients have a complete phenotype and similar RPE atrophic modifications.

## Appendix 1. Comparison of the main characteristics of the cases of posterior microphthalmos and retinitis pigmentosa associated with MFRP mutations (review of the literature).

To access the data, click or select the words “Appendix 1.” This will initiate the download of a compressed (pdf) archive that contains the file. BCVA: best-corrected visual acuity; ONH: optic nerve head. BE: both eyes. RE: right eye. LE: left eye. NLP: no light perception. HM: hand motion. RPE: retinal pigmented epithelium. RP: retinitis pigmentosa. ERG: electroretinogram. n.r.: not reported in the source paper. * this frameshift mutation shortens a set of 7 consecutive cytosines running from position 492 to 498 in exon 5 and has been named alternatively c.492delC or c.498delC in previous publications. Other two cases sharing mutation c.492delC in compound heterozygosis with a different MFRP mutation [6] were not included in this table because no clinical sign of retinal degeneration was reported.
